# Severe Pneumonia as a Complication of Scrub Typhus: A Case Report From an Endemic Region

**DOI:** 10.7759/cureus.89084

**Published:** 2025-07-30

**Authors:** Kyaw Zin Aung, Ibrahim Hassan, Ei E Cho, Phyo S Bo, Randhir K Sah

**Affiliations:** 1 Internal Medicine, Kulhudhuffushi Regional Hospital, Kulhudhuffushi City, MDV; 2 Emergency Department, Kulhudhuffushi Regional Hospital, Kulhudhuffushi City, MDV

**Keywords:** doxycycline and azithromycin treatment, endemic region, eschar, immunosuppression, scrub typhus, severe pneumonia

## Abstract

Scrub typhus, caused by *Orientia tsutsugamushi*, transmitted through bites from infected chiggers (larval mites), is a common infection in the Asia-Pacific region, including the Maldives, and typically presents with fever with myalgia, rash, eschar, and internal organ involvement. Pulmonary complications like severe pneumonia are less common but can be life-threatening. We report a case of a 51-year-old woman with underlying hypertension and rheumatoid arthritis on immunosuppressive therapy, who presented with high-grade fever, dry cough, and worsening shortness of breath. Physical examination revealed an eschar on her left thigh and crackles at both lung bases. Chest imaging showed bilateral ground-glass opacities consistent with interstitial pneumonia. Serological testing confirmed scrub typhus infection. She was treated with a combination therapy of doxycycline and azithromycin, along with supportive care in the ICU, resulting in full recovery. This case highlights the need to consider scrub typhus in patients with severe pneumonia from endemic areas, especially in those with underlying health conditions, to ensure timely diagnosis and treatment, which is critical for reducing complications of pneumonia and morbidity.

## Introduction

Scrub typhus is a sudden-onset feverish illness caused by the bacterium *Orientia tsutsugamushi*, which is gram-negative and must live inside cells. Humans contract it through bites from infected chigger larvae, the immature form of trombiculid mites. This disease continues to pose a major public health challenge in the Asia-Pacific region and is a frequent cause of fever in areas where it is endemic [1]. In the summer of 2002, the Maldives experienced an outbreak of scrub typhus, with the Ministry of Health documenting 168 suspected and confirmed cases, resulting in 10 fatalities [2]. The disease is particularly common in rural and resource-poor areas, where delayed diagnosis and limited healthcare access, including testing policies, often hinder timely treatment [3].

Clinically, it usually manifests with nonspecific symptoms like fever, muscle pain, rash, headache, swollen lymph nodes, and red eyes. In severe cases, the disease can progress to affect multiple organ systems, leading to complications such as acute respiratory distress syndrome (ARDS), pneumonia, acute liver damage, acute kidney injury, and meningoencephalitis [4]. Lung involvement is a known but rare complication of scrub typhus infection. Lung damage can range from mild to severe interstitial pneumonia, acute noncardiogenic pulmonary edema, and alveolar bleeding, often due to widespread vasculitis and damage to the endothelium [5,6].

Radiological findings often affect the lower lung zones and may show bilateral reticulonodular opacities, ground-glass changes, consolidation, septal thickening, and enlarged hilar lymph nodes. The presence of interstitial pneumonia is considered an indicator of disease severity and may suggest poor outcomes [7]. Delayed recognition due to the nonspecific nature of early symptoms and lack of clinical suspicion can lead to life-threatening complications and extended stays in the intensive care unit (ICU). Therefore, early diagnosis and appropriate antibiotic treatment are crucial. Doxycycline remains the primary treatment for scrub typhus; however, other antibiotics such as azithromycin, tetracycline, and chloramphenicol have also demonstrated clinical efficacy [8]. In more severe cases, combination therapy with doxycycline and azithromycin may be considered to enhance treatment outcomes [9].

## Case presentation

A 51-year-old Maldivian housewife initially presented to the outpatient department of Shaviyani (SH) Atoll Hospital with a seven-day history of high-grade fever, generalized myalgia, anorexia, multiple episodes of vomiting, and a dry cough associated with progressive shortness of breath. Despite multiple outpatient visits and initial management with oral amoxicillin and supportive therapy, her symptoms failed to improve, prompting hospital admission for intravenous ceftriaxone, oxygen supplementation, and close observation.

After three days of inpatient care, her respiratory symptoms worsened, and she was referred to Kulhudhuffushi Regional Hospital for further evaluation and treatment. Her past medical history included stage II hypertension, for which she was on losartan 50 mg once daily, and rheumatoid arthritis managed with tofacitinib 5 mg daily, hydroxychloroquine 200 mg daily, and methotrexate 15 mg weekly.

**Figure 1:**
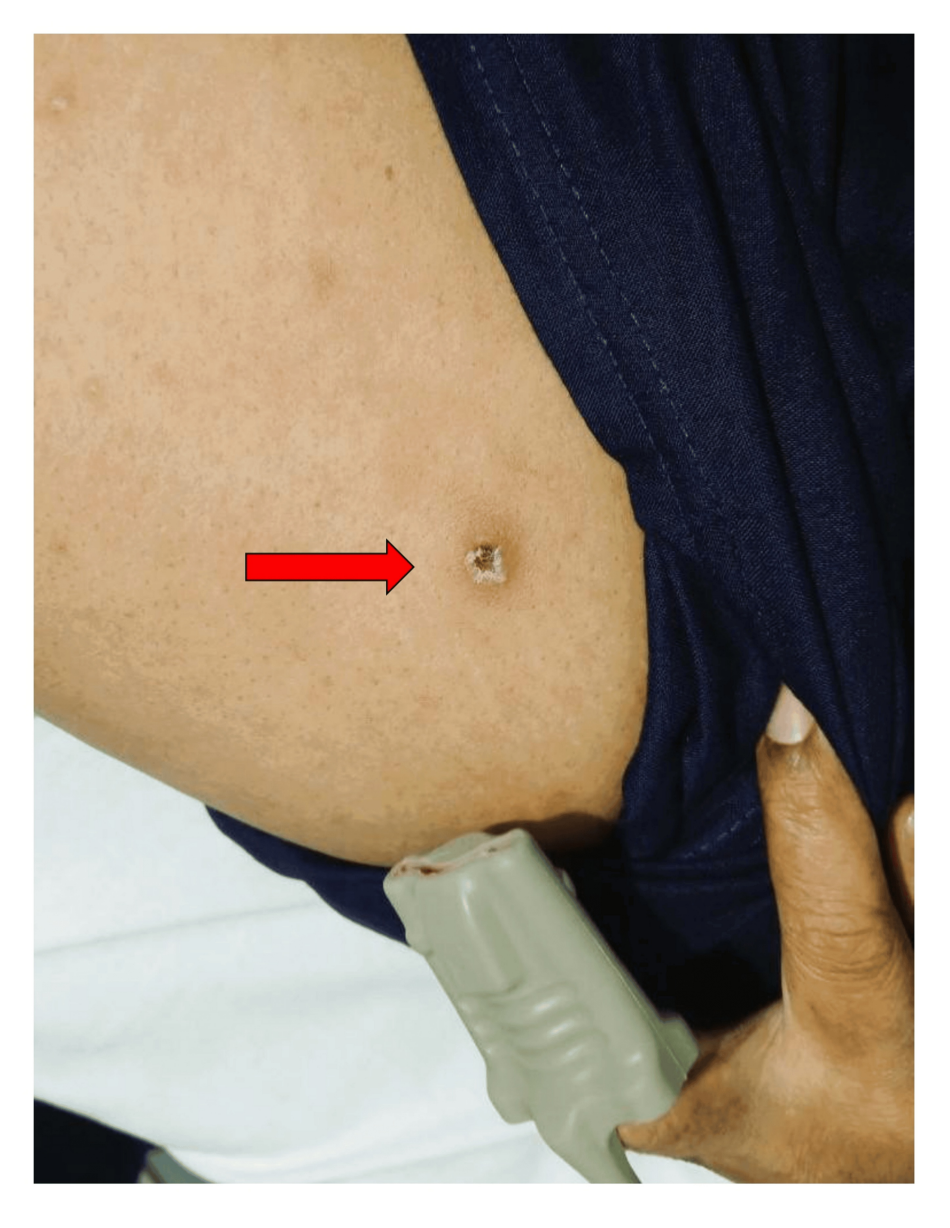
Oval-shaped eschar (red arrow) measuring approximately 1 × 0.8 cm with a black necrotic center and erythematous border on the left thigh

Upon arrival at the emergency department, the patient appeared acutely ill and in marked respiratory distress. Her vital signs were as follows: blood pressure 100/60 mmHg, respiratory rate 36 breaths/min, heart rate 110 beats/min, and oxygen saturation 85% on room air, improving to 95% with oxygen via facemask at 7 L/min. Auscultation revealed bilateral basal coarse crepitations. Jugular venous pressure was not elevated. Neurological examination showed a Glasgow Coma Scale score of 15/15, and no signs of meningism were present [10]. Abdominal examination revealed no hepatosplenomegaly. A solitary, painless, oval-shaped eschar measuring approximately 1 × 0.8 cm with a black necrotic center and erythematous border was identified on the left thigh (Figure 1).

**Figure 2:**
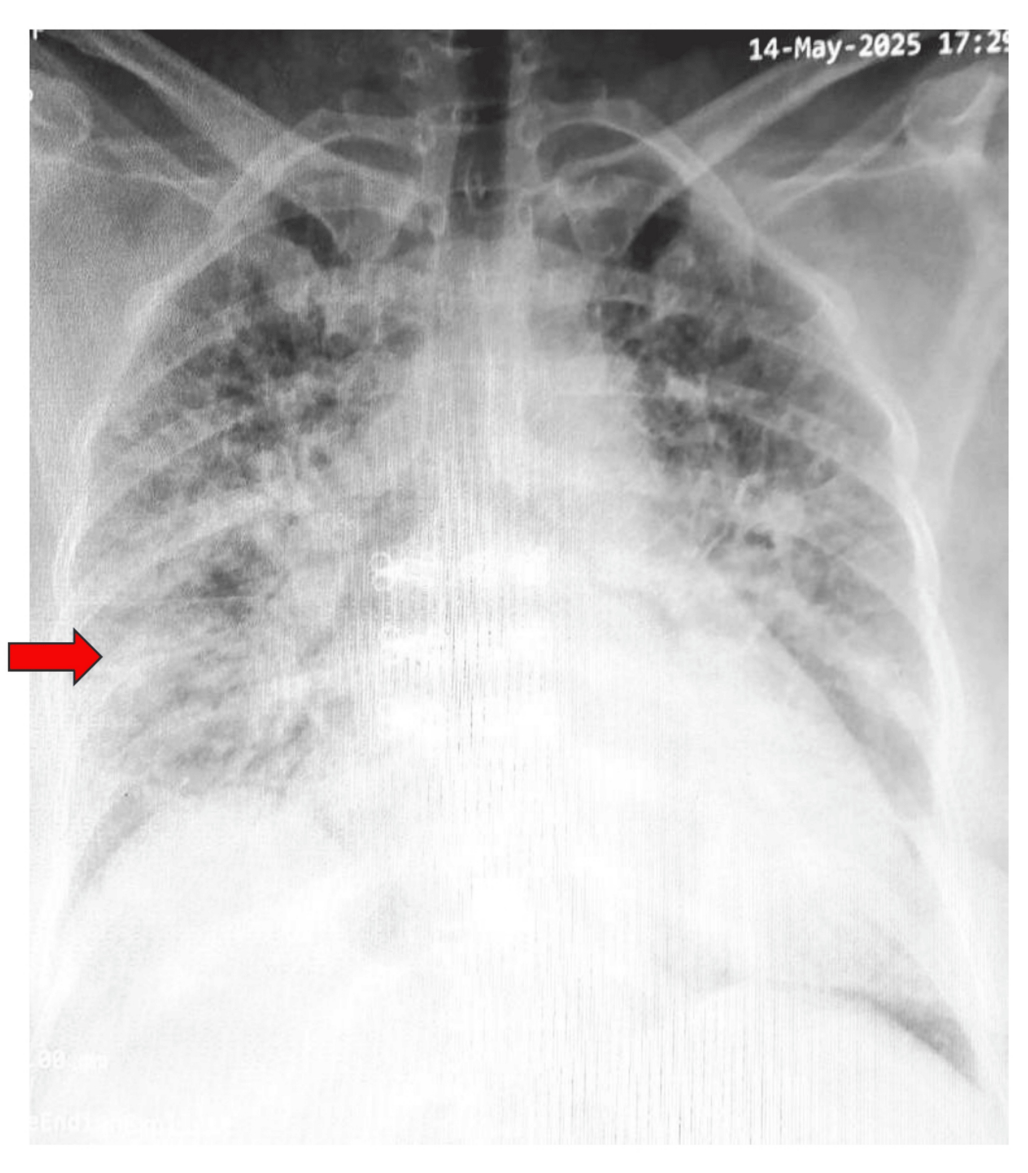
Chest X-ray on admission demonstrated bilateral patchy opacities in the middle and lower lung zones (red arrow)

Initial chest radiograph showed patchy opacities in both lower and middle lung zones (Figure 2).

**Figure 3:**
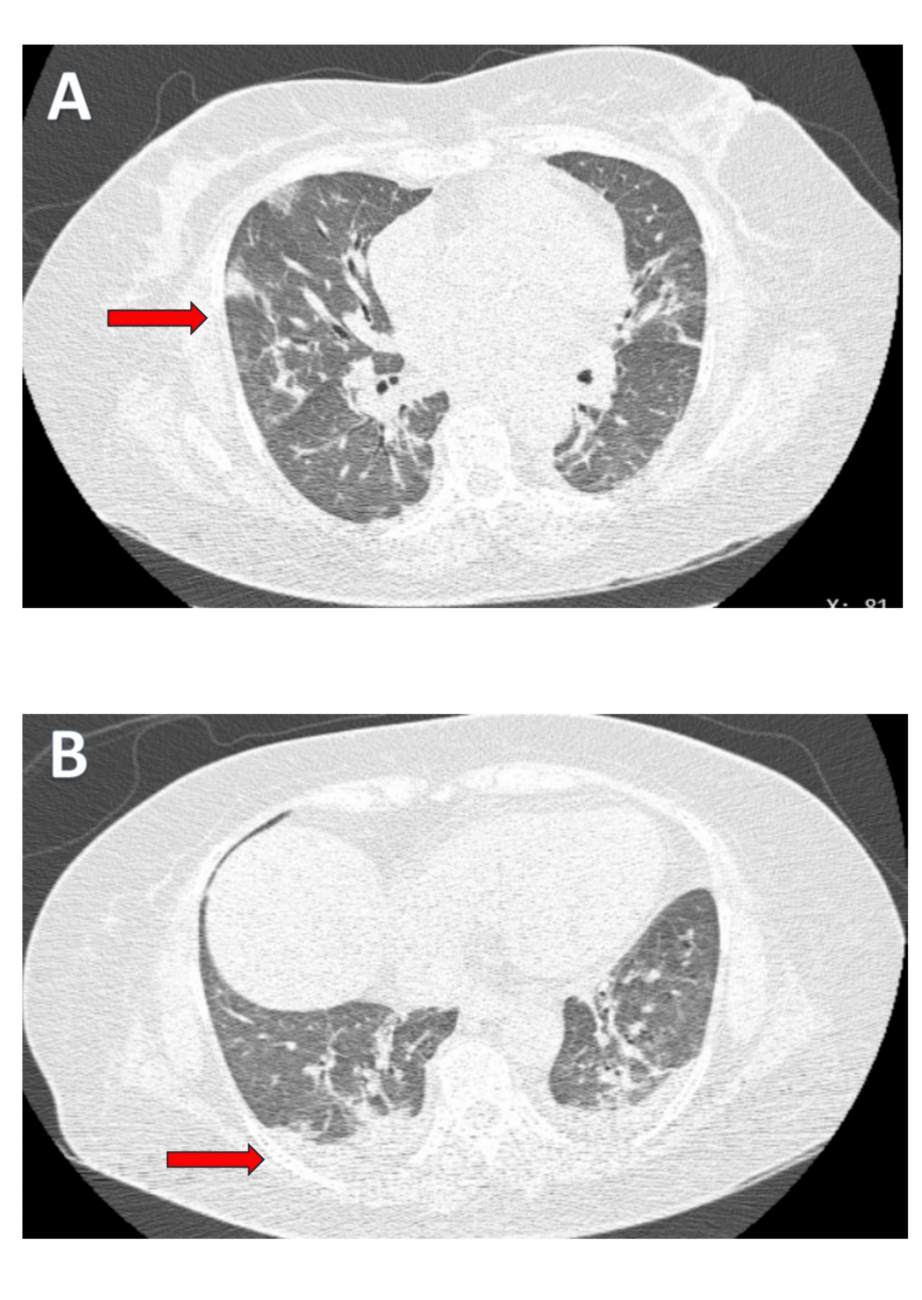
High-resolution CT (HRCT) of the chest demonstrating multiple patchy ground-glass opacities and bilateral small pleural effusions (A) Bilateral, multiple small patchy ground-glass opacities with a predominantly peripheral distribution (red arrow). (B) Bilateral small pleural effusions accompanied by ground-glass opacities (red arrow).

High-resolution computed tomography (HRCT) of the chest performed on day 1 admission revealed bilateral small pleural effusions along with multiple peripheral ground-glass opacities predominantly in the lower lobes (Figures 3A, 3B).

Laboratory investigations showed elevated inflammatory markers, and serologic testing returned positive for scrub typhus IgM by ELISA. Dengue non-structural protein 1 (NS1) and IgM were negative. Blood and sputum cultures were sent and later returned negative.

In view of the confirmed diagnosis of scrub typhus and the patient’s immunosuppressive status, she was started on oral doxycycline 100 mg twice daily and azithromycin 500 mg once daily in combination with intravenous meropenem and hydrocortisone. Disease-modifying antirheumatic drugs (DMARDs) and losartan were temporarily withheld. The patient was transferred to the intensive care unit (ICU) for close monitoring.

Over the following days, her clinical status steadily improved with declining C-reactive protein (CRP) levels and respiratory symptoms. Repeat HRCT chest on day 6 of admission showed marked radiological improvement, although some residual ground-glass opacities persisted. Blood and sputum cultures remained negative throughout her hospital stay.

She was discharged in stable condition with oral doxycycline to complete a 14-day course. Laboratory and arterial blood gas (ABG) parameters were systematically assessed during the patient’s hospitalization, including the intensive care unit stay, as well as at subsequent follow-up visits. The findings, summarized in Table 1, illustrate a clear trend of gradual improvement in these clinical markers, correlating with the patient’s overall recovery. At her follow-up visit, she showed complete clinical recovery; C-reactive protein (CRP) had normalized (<5 mg/L), and chest X-ray demonstrated full resolution of the previously noted opacities. Her antihypertensive and immunosuppressive therapies were safely restarted.

**Table 1:** Summary of laboratory investigations at admission and on subsequent follow-up days Hb: hemoglobin; WBC: white blood cell count; ANC: absolute neutrophil count; ALC: absolute lymphocyte count; CRP: C-reactive protein; ALT: alanine aminotransferase; AST: aspartate aminotransferase; ALP: alkaline phosphatase; Cr: creatinine; BUN: blood urea nitrogen; PaO₂: partial pressure of oxygen; PaCO₂: partial pressure of carbon dioxide. Day 1 refers to the day of admission; subsequent days follow accordingly.

Parameters	Day 1	Day 3	Day 5	Day 7	Day 14	Reference
Hb (g/dL)	9.7	10.1	9.3	9.6	10.4	10.6-13.5
WBC (×10⁹/L)	13.09	17.74	11.9	12.45	5.7	4-11.5
ANC (×10⁹/L)	11.8	13.2	7.6	9	2.4	2-7.15
ALC (×10⁹/L)	0.7	2.4	2.8	2.2	2.6	1.16-3.18
Platelet (×10⁹/L)	203	236	341	412	372	150-450
CRP (mg/L)	365.2	77.9	31.7	15.9	<5	<10
Total bilirubin (mg/dL)	0.6	0.6	0.5	0.4	0.4	0.2-1.3
ALT (U/L)	69	48	42	22	18	<35
AST (U/L)	94	46	40	24	18	14-36
ALP (U/L)	169	152	120	89	69	38-126
Cr (mg/dL)	1.2	1	1.1	0.7	0.6	0.52-1.04
BUN (mg/dL)	20.1	30.8	24.6	21.5	18.4	7-17
pH	7.44	7.46	7.4	7.49	7.42	7.35-7.45
PaO_2_ (mmHg)	60	76.7	86.6	83.5	88.4	83-108
PaCO_2_ (mmHg)	32	39.7	38	34.1	38.2	32-48

## Discussion

Scrub typhus is an important public health problem in many parts of the Asia-Pacific region, especially within the area known as the "scrub typhus triangle." Although the Maldives is not typically included in this triangle, its close location to South Asia and similar environmental and ecological conditions make the presence of the disease likely. The burden of scrub typhus is high in the Maldives, and it may be underdiagnosed or underreported in the Maldives [1,2]. Globally, more than one billion people live in areas where they are at risk of infection. If not treated early, scrub typhus can lead to serious complications and may result in death, with fatality rates reaching up to 30% in severe cases [3]. This case highlights the importance of early detection of scrub typhus through detailed history taking, careful physical examination, particularly the identification of an eschar, and timely laboratory testing for antibodies. Recognizing the disease early and starting proper treatment can lead to better outcomes and help prevent serious complications, especially in patients with immunosuppressant conditions. In immunocompromised patients, the clinical presentation of scrub typhus may be atypical, with eschars often absent or difficult to detect. The suppressed immune response can delay symptom onset and obscure key diagnostic features, increasing the risk of missed or late diagnosis and may allow for more rapid progression to severe disease. This patient is also more susceptible to severe disease progression due to impaired pathogen clearance.

Scrub typhus manifests across a broad clinical spectrum, ranging from mild symptoms such as fever, myalgia, rash, lymphadenopathy, and characteristic eschar, to severe complications including pneumonia, acute respiratory distress syndrome (ARDS), and multiorgan failure. The causative agent, *Orientia tsutsugamushi*, predominantly invades vascular endothelial cells, precipitating widespread systemic vasculitis and perivascular inflammation that contribute to multisystem involvement. Pulmonary manifestations are common and may vary from subtle interstitial infiltrates to fulminant interstitial pneumonia, ARDS, and pulmonary hemorrhage [4-7]. In this case, the presence of high-grade fever with cough unresponsive to initial empirical antibiotics, an eschar on the left thigh, chest imaging showing multiple small ground-glass opacities in both lung fields, and a positive antibody test helped in the early detection of scrub typhus-associated lung involvement at tertiary care hospital. Inadequate or delayed diagnosis and treatment correlate strongly with increased morbidity and respiratory failure. Therefore, early identification of pulmonary involvement is important to guide timely therapeutic intervention and improve patient outcomes.

Doxycycline remains the mainstay of therapy for scrub typhus and is effective even in patients with severe pulmonary complications [8]. In cases with poor initial response or severe systemic involvement, combination therapy has been explored. Recent evidence from a multicenter randomized controlled trial (INTREST Trial) indicates that intravenous doxycycline combined with azithromycin offers superior outcomes compared to monotherapy with either drug alone [9]. In this case, the patient was immunocompromised due to long-term use of DMARDs for rheumatoid arthritis. Given the risk of secondary bacterial infections in an immunosuppressed host, empirical treatment with intravenous meropenem was initiated alongside oral doxycycline and azithromycin. Notably, the patient showed marked clinical improvement within 72 hours of starting doxycycline and azithromycin, with resolution of fever, improvement in respiratory status, and a significant downward trend in CRP levels. This rapid response highlights the critical role of early doxycycline and azithromycin administration in reducing disease progression and preventing complications such as ARDS.

## Conclusions

Community-acquired pneumonia is commonly seen in patients who are immunosuppressed due to underlying conditions or comorbidities. However, pneumonia caused by scrub typhus is relatively uncommon and can easily be mistaken for other types of pneumonia, such as bacterial, viral, or fungal infections, particularly in immunocompromised individuals. In this case, maintaining a broad differential diagnosis and considering atypical pathogens are crucial. It is therefore important to include scrub typhus in the differential diagnosis of pneumonia unresponsive to the first-line antimicrobial therapy, as awareness of regional disease prevalence and subtle clinical indicators can facilitate early identification and appropriate management, ultimately improving outcomes in this high-risk population.
